# Strong Tracking Spherical Simplex-Radial Cubature Kalman Filter for Maneuvering Target Tracking

**DOI:** 10.3390/s17040741

**Published:** 2017-03-31

**Authors:** Hua Liu, Wen Wu

**Affiliations:** Ministerial Key Laboratory of JGMT, Nanjing University of Science and Technology, Nanjing 210094, China; peterliuh@126.com

**Keywords:** maneuvering target tracking, spherical simplex-radial rule, cubature Kalman filter, fading factor, strong tracking filter

## Abstract

Conventional spherical simplex-radial cubature Kalman filter (SSRCKF) for maneuvering target tracking may decline in accuracy and even diverge when a target makes abrupt state changes. To overcome this problem, a novel algorithm named strong tracking spherical simplex-radial cubature Kalman filter (STSSRCKF) is proposed in this paper. The proposed algorithm uses the spherical simplex-radial (SSR) rule to obtain a higher accuracy than cubature Kalman filter (CKF) algorithm. Meanwhile, by introducing strong tracking filter (STF) into SSRCKF and modifying the predicted states’ error covariance with a time-varying fading factor, the gain matrix is adjusted on line so that the robustness of the filter and the capability of dealing with uncertainty factors is improved. In this way, the proposed algorithm has the advantages of both STF’s strong robustness and SSRCKF’s high accuracy. Finally, a maneuvering target tracking problem with abrupt state changes is used to test the performance of the proposed filter. Simulation results show that the STSSRCKF algorithm can get better estimation accuracy and greater robustness for maneuvering target tracking.

## 1. Introduction

Maneuvering target tracking has drawn increasing attention because of its widespread application in areas such as radar tracking, aircrafts surveillance, and spacecraft orbit control [1,2]. For maneuvering target tracking, many algorithms are developed and grouped into two types. One type is to improve the accuracy of the motion model, such as multiple-model (MM) methods [3], optimization of multiple model neural filter [4], current statistical (CS) model [5,6], and so on. The other type is to detect the target maneuverability and then to cope with it effectively, such as strong tracking filter (STF) [7], tracking algorithm based on maneuvering detection [8], and so on. In these methods, the performance of the filter is an important factor affecting the performance of these methods. Therefore, improving the accuracy of the filter is also a useful method to improve the performance of maneuvering target tracking. Thus, a large number of nonlinear filters have been developed. Among these algorithms, the extended Kalman filter (EKF) [9] is one of the earliest and most widely used nonlinear filters. The EKF uses a linearization technique, based on the first-order Taylor series expansion, and approximates the nonlinear system. However, EKF has some limitations, such as complex Jacobian matrix calculations and poor accuracy in estimating the states of the strongly nonlinear system.

As better alternatives to the EKF, many nonlinear filters based on the idea of Bayesian theory have been proposed. One popular approach for the nonlinear non-Gaussian filtering problem is to use sequential Monte Carlo methods. The most famous method is known as particle filter (PF) [10,11,12,13]. The key idea of PF is to represent the posterior distribution by a set of random samples and to calculate estimates based on these samples and weights. Although the PF can provide good performance, the computational cost is very high and suffers from the curse of the dimensionality problem. These shortcomings restrict their applications in a real-time system. A different approach for nonlinear filtering is based on the point-based filtering technique that approximates intractable integrals encountered by a set of deterministically sampled points. Compared with the Monte Carlo numerical integration that relies on randomly sampled points, the deterministic point-based method has lower computational complexity with high accuracy. The type of filter includes the unscented Kalman filter (UKF) [14], Gauss-Hermite filter (GHF) [15], central difference filter (CDF) [16], etc. Among these methods, the well-known filter is UKF. The UKF uses unscented transform (UT) to capture the mean and covariance of a Gaussian density. It is shown that the UKF has better performance than the EKF. Besides its higher approximation accuracy, this UKF can avoid the cumbersome evaluation of Jacobian and Hessian matrices, making the algorithm easier to implement. Nevertheless, the unscented transform of the UKF is potentially unstable [17], which restricts its practical applications. Apart from the aforementioned filters, the cubature Kalman filter (CKF) has been proposed [17,18] by Arasaratnam and Haykin. Making use of the third-degree spherical-radial cubature rule, the CKF is reported to be more flexible in implementation form and more stable than UKF. In addition, Jia et al. [19] proposed the high-degree CKF where the number of sample points increases rapidly with the increase of the degree or state dimension. To further improve estimation accuracy with low complexity, a new nonlinear filter named spherical simplex-radial cubature filter (SSRCKF) is developed in [20]. The new class of CKF is based on the simplex spherical radial (SSR) rule, which improves the accuracy of CKF with only two more cubature points necessary.

Although the SSRCKF can achieve good accuracy in tracking non-maneuvering or weak maneuvering targets, it may lose the tracking ability to the abrupt state change when the system reaches the stable state. This is because the reaction of the gain matrix is delayed to the sudden change of the prediction error. To tackle the problem mentioned above, a new algorithm called strong tracking spherical simplex-radial cubature Kalman filter (STSSRCKF) is proposed in this paper. The STSSRCKF is developed based on the combination of strong tracking filter (STF) [7,21,22] and SSRCKF. The new algorithm using the strong tracking idea and the fading factor based on the residual to modify the prior covariance matrix quickly. Thus, the gain of the filter can be adjusted in real time to enhance tracking capacity for the maneuvering target. In addition, the algorithm can also keep a normal tracking accuracy for weak maneuvering targets. Compared with the STF, strong tracking unscented Kalman filter (STUKF) [23], strong tracking cubature Kalman filter (STCKF) [24] and SSRCKF, the proposed algorithm has a good accuracy and robust advantage over a wide range of maneuver. The performance of the proposed filter is demonstrated by the simulation.

The remainder of this paper is organized as follows. The overview of the background theory is presented in Section 2. The proposed algorithm is developed in Section 3. Simulation results and performance comparisons are presented in Section 4. Finally, conclusions are provided in Section 5.

## 2. A Review of UKF and CKF

The nonlinear discrete-time system is represented by

(1) $$\left\{ \begin{array}{l} x_{k}=f\left(x_{k-1}\right)+w_{k-1}\\ z_{k}=h\left(x_{k}\right)\;+v_{k} \end{array}\right.$$

where k∈N denotes discrete time, f(⋅) represents the nonlinear function, h(⋅) represents the measurement function. $x_{k}\in R^{n}$ is the state vector of system, $z_{k}\in R^{m}$ is the measurement, $w_{k}\in R^{n}$ is the process noise vector, and $v_{k}\in R^{m}$ is the measurement noise vector. The process $w_{k}$ and measurement noise $v_{k}$ are uncorrelated zero-mean Gaussian white sequences and have zero cross-correlation with each other, represented as $w_{k}\sim N(0,Q_{k})$ and $v_{k}\sim$ $N(0,R_{k})$, respectively.

Under the Gaussian assumption in the Bayesian filtering framework, the key problem of the nonlinear filtering problem is to calculate the multi-dimensional integrals. However, in most cases, the multi-dimensional integrals cannot be solved analytically. As a result, several approximation methods have been proposed, such as the unscented transformation (UT) and the cubature rule.

The UT with 2n+1 the sigma points $\chi_{i}$ and corresponding weights is chosen as

(2) $$\left\{ \begin{array}{l} \chi_{0}=x_{k|k}\\ \chi_{i}=x_{k|k}+{\left[\sqrt{(n+\lambda )P_{k|k}}\right]}_{i}\;(i=1,\cdots ,n)\\ \chi_{i}=x_{k|k}-{\left[\sqrt{(n+\lambda )P_{k|k}}\right]}_{i}\;(i=n+1,\cdots ,2n)\\ \omega_{m}^{\left(0\right)}=\lambda /(n+\lambda )\;\\ \omega_{c}^{\left(0\right)}=\lambda /(n+\lambda )+(1-\alpha^{2}+\beta )\\ \omega_{m}^{\left(i\right)}=\omega_{c}^{\left(i\right)}=1/2(n+\lambda )(i=1,\cdots ,2n) \end{array}\right.$$

where ${\left[P_{k|k}\right]}_{i}$ is the ith column of the matrix square root of $P_{k|k}$, n is the dimension of state. $\lambda =\alpha^{2}(n+\kappa )-n$ is the scaling parameter; α determines the spread of the sigma points around $x_{k|k}$. The positive constants β and κ are used as parameters of the method.

The third-degree cubature rule with 2n cubature points and weights is given by:

(3) $$\left\{ \begin{array}{l} \chi_{i}=x_{k|k}+{\left[\sqrt{(n/2)P_{k|k}}\right]}_{i}\;(i=1,\cdots ,n)\\ \chi_{i}=x_{k|k}-{\left[\sqrt{(n/2)P_{k|k}}\right]}_{i}\;(i=n+1,\cdots ,2n)\\ \omega_{m}^{\left(i\right)}=\omega_{c}^{\left(i\right)}=1/2n\;(i=1,\cdots ,2n) \end{array}\right.$$

As indicated above, the main difference between the UT used in UKF and the third-degree cubature rule used in CKF is that the UT has one more point in the center with a tune parameter κ. If the parameter κ is set to zero, the sigma points set will evolve into the cubature points set and the UKF becomes identical to the CKF. For UKF, the scaling parameter κ is always set to n−3. Based on this point, for high-dimensional problems (n>3), it will lead to the negative weight of the center point. The presence of the negative weight may lead the covariance matrix to become non-positively defined. Thus, the cubature rule is more stable than the UT. In summary, the CKF is virtually a special case of UKF and the CKF has better numerical stability than UKF.

## 3. Strong Tracking Spherical Simplex-Radial Cubature Kalman Filter

The heart of the spherical simplex cubature Kalman filter is the spherical-radial cubature rule. The spherical-radial cubature rule does not approximate the nonlinear function, but it can approximate the integral of the form (nonlinear function × Gaussian) using weighted quadrature point sets. The integral with the standard Gaussian distribution N(x;0,I) can be approximated by the quadrature

(4) $$\int_{{\mathbb{R}}^{n}}f(x)N(x;0,I)dx\approx \sum_{i=1}^{m}\omega_{i}f(\gamma_{i})$$

where m is the total number of quadrature points in the state-space ${\mathbb{R}}^{n}$, ${\left\{\gamma_{i},\omega_{i}\right\}}_{i}^{m}$ is a set of quadrature points and corresponding weights. The general Gaussian integral $\int_{{\mathbb{R}}^{n}}f(x)N(x;\hat{x},P)dx$ can be approximated by the following transformation

(5) $$\begin{array}{cc} \int_{{\mathbb{R}}^{n}}f(x)N(x;\hat{x},P)dx & =\int_{{\mathbb{R}}^{n}}f(\sqrt{P}x+\hat{x})N(x;0,I)dx\\ & \approx \sum_{i=1}^{m}\omega_{i}f(\sqrt{P}\gamma_{i}+\hat{x}) \end{array}$$

The computational complexity of the numerical integration is proportional to the number of quadrature points, and the accuracy of the numerical integration rule is usually assessed by the polynomial approximation degrees.

### 3.1. Review of the Third-Degree Spherical Simplex-Radial Cubature Rule

The SSRCKF algorithm has the same structure as the general Gaussian approximation filters, such as the CKF, but uses the third-degree spherical simplex-radial cubature rule to calculate the Gaussian weight integral $I(f)=\int_{R^{n}}f(x)N(x;0,I)dx$. By using the spherical simplex-radial cubature rule, the SSRCKF method can get more accurate estimation than CKF. In the third-degree spherical simplex-radial cubature rule, the following integral is considered [19]:

(6) $$I(f)=\int_{R^{n}}f(x)\exp(-x^{T}x)dx$$

where f(⋅) is arbitrary nonlinear function, ${\mathbb{R}}^{n}$ is the integral domain. To calculate the above integral, let x=rs $\left(s^{T}s=1,r=\sqrt{x^{T}x}\right)$. Equation (6) can be transformed into the spherical-radial coordinate system

(7) $$I(f)=\int_{0}^{\infty }\int_{U_{n}}f(rs)r^{n-1}\exp(-r^{2})d\sigma (s)\;dr$$

where $s={\left[s_{1},s_{2},\cdots ,s_{n}\right]}^{T}$, $U_{n}=\{s\in {\mathbb{R}}^{n}:$ $s_{1}^{2}+s_{2}^{2}$ $+\cdots +s_{n}^{2}=1\}$ is the spherical surface, and σ(⋅) is the area element on $U_{n}$. Then, the Equation (7) can be decomposed into the spherical integral $S(r)=\int_{U_{n}}f(rs)d\sigma (s)$ and the radial integral $I(f)=\int_{0}^{\infty }S(r)r^{n-1}\exp(-r^{2})dr$.

#### 3.1.1. Spherical Simplex Rule

As can be seen from the literature [25], the spherical integral $\int_{U_{n}}f(rs)d\sigma (s)$ can be approximated by the transformation group of the regular n-simplex with vertices $a_{j}$. $P_{0}$ The third-degree spherical simplex rule with 2n+2 quadrature points is given by

(8) $$\begin{array}{cc} S(r) & =\frac{A_{n}}{2(n+1)}\sum_{j=1}^{n+1}\left(f(ra_{j})+f(-ra_{j})\right)\\ & =\sum_{j=1}^{N_{s}}\omega_{s,j}f(ry_{j}) \end{array}$$

where $A_{n}=2\sqrt{\pi^{n}}/\Gamma^{n}(1/2)$, $N_{s}=2n+2$.

#### 3.1.2. Radial Rule

The radial integral $\int_{0}^{\infty }S(r)r^{n-1}\exp(-r^{2})dr$ can be calculated by the following moment matching equation

(9) $$\int_{0}^{\infty }S(r)r^{n-1}\exp(-r^{2})dr=\sum_{i=1}^{N_{r}}\omega_{r,i}S(r_{i})$$

where $S(r)=r^{l}$ is a monomial in r, with l an even integer. Using the moment method with the minimum number of points, the third-degree radial rule $\left(N_{r}=1\right)$ can be derived. From Equation (9) we can obtain the moments’ equations as

(10) $$\left\{ \begin{array}{l} \omega_{r,1}r_{1}^{0}=\frac{1}{2}\Gamma (\frac{n}{2})\\ \omega_{r,1}r_{1}^{2}=\frac{1}{2}\Gamma (\frac{n+2}{2})=\frac{n}{4}\Gamma (\frac{n}{2}) \end{array}\right.$$

By solving Equation (10), the points and weights for the third-degree radial rule are given by

(11) $$\left\{ \begin{array}{l} r_{1}=\sqrt{n/2}\\ \omega_{r,1}=\Gamma (n/2)/2 \end{array}\right.$$

#### 3.1.3. Spherical Simplex-Radial Rule

By using Equations (7), (8) and (11), the third-degree spherical simplex-cubature rule ($N_{r}=1$, $N_{s}=2n+2$) is given by

(12) $$\begin{array}{l} \int_{R^{n}}f(x)N(x,0,I)dx\\ =\frac{1}{\sqrt{\pi^{n}}}\int_{R^{n}}f(\sqrt{2}x)\exp(-x^{T}x)dx\\ \approx \frac{1}{\sqrt{\pi^{n}}}\sum_{i=1}^{N_{r}}\sum_{j=1}^{N_{s}}\omega_{r,i}\omega_{s,j}f(\sqrt{2}r_{i}s_{j})\\ =\frac{1}{2(n+1)}\times \left(\sum_{j=1}^{n+1}f(\sqrt{n}a_{j})+\sum_{j=n+2}^{2n+2}f(-\sqrt{n}a_{j}))\right)\\ =\sum_{k=1}^{m}\omega_{k}f(\xi_{k}) \end{array}$$

where m=2n+2, $\xi_{k}=$ $\sqrt{n}{\left[a,-a\right]}_{k}$ and $\omega_{k}=$ 1/(2n+2) are the corresponding weights.

The steps of SSRCKF algorithm for the nonlinear system can be found in the literature [17].

### 3.2. Strong Tracking Filter

To improve the performance of EKF, a concept of STF was proposed by Zhou and Frank [7]. They proved that a filter can obtain the strong tracking estimation of the state can have the strong tracking performance only if the filter satisfies the orthogonal principle [7]. In strong tracking, the time-varying suboptimal fading factor is incorporated, which online adjusts the covariance of the predicted state. In this way, the algorithm has the ability to track abrupt state change and strong robustness against mode uncertainties. The algorithm has the following steps [21]:

(13) $$\begin{array}{l} {\hat{x}}_{k|k-1}=f_{k}({\hat{x}}_{k-1|k-1})\\ P_{k|k-1}=\lambda_{k}F_{k|k-1}P_{k}F_{k|k-1}^{T}+Q_{k}\\ K_{k}=P_{k|k-1}H_{k}^{T}{\left(H_{k}P_{k|k-1}H_{k}^{T}+R_{k}\right)}^{-1}\\ {\hat{x}}_{k|k}={\hat{x}}_{k|k-1}+K_{k}(z_{k}-h({\hat{x}}_{k|k-1}))\\ P_{k|k}=[I-K_{k}H_{k}]P_{k|k-1} \end{array}$$

where $F_{k|k-1}$ and $H_{k}$ are the process matrix and measure matrix, respectively. The suboptimal time-varying fading factor $\lambda_{k}$ is given by

(14) $$\lambda_{k}=\{\begin{array}{cc} c_{k}, & c_{k}\geq 1\\ 1, & c_{k}<1 \end{array},c_{k}=\frac{\mathrm{tr}[N_{k}]}{\mathrm{tr}[M_{k}]}$$

(15) $$N_{k}=V_{k}-H_{k}Q_{k-1}H_{k}^{T}-\beta R_{k}$$

(16) $$M_{k}=H_{k}F_{k}P_{k-1|k-1}F_{k}^{T}H_{k}^{T}$$

(17) $$V_{k}=\{\begin{array}{cc} v_{0}v_{0}^{T} & k=0\\ \frac{\rho V_{k-1}+v_{k}v_{k}^{T}}{1+\rho } & k\geq 1 \end{array}$$

where tr[⋅] is the trace operation, $v_{k}=z_{k}-{\hat{z}}_{k|k-1}$ denotes the measurement residual vector; β≥1 is the softening factor, which can improve the smoothness of state estimation; 0<ρ≤1 is the forgetting factor. In generally, the parameters β and ρ are chosen as 4.5 and 0.95, respectively [26,27].

### 3.3. Equivalent Expression of the Fading Factor

As we know, STF need calculate the linearization of the nonlinear measurement matrix (Hessian matrix). However, SSRCKF is not necessary to compute the Hessian matrix. So we give the equivalent expression of STF, which need not calculate the Hessian matrix. Suppose $P_{k|k-1}^{l}$ is the state error covariance matrix before introducing fading factor, $P_{zz,k|k-1}^{l}$ is the measurement covariance matrix and $P_{xz,k|k-1}^{l}$ is cross-covariance matrix, Equations (15) and (16) have the following equivalent expressions:

(18) $$N_{k}=V_{k}-{\left(P_{xz,k|k-1}^{l}\right)}^{T}{\left(P_{k|k-1}^{l}\right)}^{-1}Q_{k-1}{\left(P_{k|k-1}^{l}\right)}^{-1}(P_{xz,k|k-1}^{l})-\beta R_{k}$$

(19) $$M_{k}=P_{zz,k|k-1}^{l}-V_{k}+N_{k}+(\beta -1)R_{k}$$

The new fading factor can be obtained through Equations (14) and (17)–(19). It can be verified from Equations (18) and (19) that the calculation of suboptimal fading factor in the Equation expression does not need to compute any Jacobian matrix.

### 3.4. Steps of the STSSRCKF

Based on the previous sections, the strong tracking spherical-simplex cubature Kalman filtering (STSSRCKF) can adjust the prediction error covariance matrix by introducing a suboptimal factor. Hence, the robustness and real-time tracking ability are provided in the STSSRCKF algorithm. The initial state is assumed to be Gaussian distribution with ${\hat{x}}_{0|0}$ and $P_{0|0}$. The computation steps of the third-degree strong tracking spherical simplex-radial cubature Kalman filter is summarized as follows:

**Step 1.** Give the state estimate ${\hat{x}}_{k-1|k-1}$ and the error covariance matrix $P_{k-1|k-1}$;

**Step 2.** State estimate prediction:

The cubature points are obtained as

(20) $$\chi_{j,k|k-1}^{l}={\hat{x}}_{k|k-1}+\mathrm{chol}(P_{k|k-1}{)}^{T}\xi_{j}$$

where chol(⋅)is the Cholesky factorization.

Propagate the cubature points, the predicted state $x_{k|k-1}$, and the predicted covariance $P_{k|k-1}^{l}$ without the fading factor are given as

(21) $$\chi_{j,k|k-1}^{*}=f(\chi_{j,k-1})$$

(22) $${\hat{x}}_{k|k-1}=\sum_{j=1}^{m}\omega_{j}\chi_{j,k|k-1}^{*}$$

(23) $$P_{k|k-1}^{l}=\sum_{j=1}^{m}\omega_{j}(\chi_{j,k|k-1}^{*}-{\hat{x}}_{k|k-1}){\left(\chi_{j,k|k-1}^{*}-{\hat{x}}_{k|k-1}\right)}^{T}+Q_{k-1}$$

where $Q_{k-1}$ is the covariance matrix of process noise.

**Step 3.** Calculation of the fading factor $\lambda_{k}$:

Using the predicted state ${\hat{x}}_{k|k-1}$ and the predicted covariance $P_{k|k-1}^{l}$, the innovation covariance $P_{zz,k|k-1}^{l}$ and the cross covariance $P_{xz,k|k-1}^{l}$ can be calculated as

(24) $$z_{j,k|k-1}^{l}=h(\chi_{j,k|k-1}^{*})$$

(25) $${\hat{z}}_{k|k-1}^{l}=\sum_{j=1}^{m}\omega_{j}z_{j,k|k-1}^{l}$$

(26) $$P_{xz,k|k-1}^{l}=\sum_{j=1}^{m}\omega_{j}(\chi_{j,k|k-1}^{l}-{\hat{x}}_{k|k-1}){\left(z_{j,k|k-1}^{l}-{\hat{z}}_{k|k-1}^{l}\right)}^{T}$$

(27) $$P_{zz,k|k-1}^{l}=\sum_{j=1}^{m}\omega_{j}(z_{j,k|k-1}^{l}-{\hat{z}}_{k|k-1}^{l}){\left(z_{j,k|k-1}^{l}-{\hat{z}}_{k|k-1}^{l}\right)}^{T}+R_{k}$$

The fading factor $\lambda_{k}$ can be calculated by using Equations (14) and (17)–(19).

**Step 4.** Measurement updating modified by the fading factor:

The modified prediction covariance $P_{k|k-1}^{\prime }$ can be updated by

(28) $$P_{k|k-1}^{\prime }=\lambda_{k}(P_{k|k-1}^{l}-Q_{k-1})+Q_{k-1}$$

By utilizing the predicted state estimate ${\hat{x}}_{k|k-1}$ and the modified predicted covariance $P_{k|k-1}^{\prime }$ with the fading factor $\lambda_{k}$, the modified predicted measurement ${\hat{z}}_{k|k-1}^{\prime }$, the modified cross covariance and the modified innovation covariance $P_{zz,k|k-1}^{\prime }$ can be calculated as follows

(29) $$\chi_{j,k|k-1}^{\prime }=\mathrm{chol}(P_{k|k-1}^{\prime })\xi_{i}+{\hat{x}}_{k|k-1}$$

(30) $$z_{j,k|k-1}^{\prime }=h(\chi_{j,k|k-1}^{\prime })$$

(31) $${\hat{z}}_{k|k-1}^{\prime }=\sum_{j=1}^{m}\omega_{j}z_{j,k|k-1}^{\prime }$$

(32) $$P_{xz,k|k-1}^{\prime }=\sum_{j=1}^{m}\omega_{j}(\chi_{j,k|k-1}^{\prime }-{\hat{x}}_{k|k-1}){\left(z_{j,k|k-1}^{\prime }-{\hat{z}}_{k|k-1}^{\prime }\right)}^{T}$$

(33) $$P_{zz,k|k-1}^{\prime }=\sum_{j=1}^{m}\omega_{j}(z_{j,k|k-1}^{\prime }-{\hat{z}}_{k|k-1}^{\prime }){\left(z_{j,k|k-1}^{\prime }-{\hat{z}}_{k|k-1}^{\prime }\right)}^{T}+R_{k}$$

**Step 5.** Estimation results:

The state estimate ${\hat{x}}_{k}$ and the covariance $P_{k}$ at time k are calculate as follows

(34) $$K_{k}=P_{xz,k|k-1}^{\prime }{\left(P_{zz,k|k-1}^{\prime }\right)}^{-1}$$

(35) $${\hat{x}}_{k|k}={\hat{x}}_{k|k-1}+K_{k}(z_{k}-{\hat{z}}_{k|k-1}^{\prime })$$

(36) $$P_{k|k}=P_{k|k-1}^{\prime }-K_{k}P_{zz,k|k-1}^{\prime }K_{k}^{T}$$

The STSSRCKF combines the advantages of STF and SSRCKF. Then the STSSRCKF has strong robustness against model uncertainties and good real-time state tracking capability [28]. Moreover, the STSSRCKF algorithm eliminates the cumbersome evaluation of Jacobian/Hessian matrices, its numerical stability and estimated accuracy are significantly improved.

## 4. Simulation and Results

The effectiveness of the proposed algorithm will be illustrated through two examples of maneuvering target tracking. Taking the root mean square error (RMSE) and accumulative RMSE (ARMSE), the study compared the STSSRCKF algorithm with the EKF algorithm and the SSRCKF algorithm.

### 4.1. Tracking Model and Measurement Model

The constant acceleration (CA) model is a common tool for tracking target modeling. The state Equation of CA model in two-dimensional case is described as follow:

(37) $$X_{k}=diag[\Phi_{CA},\Phi_{CA}]X_{k-1}+G_{CA}V_{k-1}+w_{k-1}$$

where $X_{k-1}={\left[x_{k-1},{\dot{x}}_{k-1},{\ddot{x}}_{k-1},y_{k-1},{\dot{y}}_{k-1},{\ddot{y}}_{k-1}\right]}^{T}$ is the target state at time k−1, $\left(x_{k-1},y_{k-1}\right)$, $\left({\dot{x}}_{k-1},{\dot{y}}_{k-1}\right)$ and $\left({\ddot{x}}_{k-1},{\ddot{y}}_{k-1}\right)$ represent the target position, velocity and acceleration in the x and y coordinate at time k−1, respectively; $diag[\Phi_{CA},\Phi_{CA}]$ is the state transition matrix, $G_{k-1}$ is the state input matrix, $V_{k-1}$ is the process noise, $w_{k-1}$ is zero-mean white Gaussian noise and its corresponding covariance matrix is $Q_{ca}$. $\Phi_{CA}$, $G_{k-1}$ are described as:

(38) $$\Phi_{CA}=\left[\begin{array}{ccc} 1 & T & T^{2}/2\\ 0 & 1 & T\\ 0 & 0 & 1 \end{array}\right]$$

(39) $$G_{CA}=\left[\begin{array}{c} T^{2}/2\\ T\\ 1 \end{array}\right]$$

where T is the sampling interval.

In radar tracking system, the target motion is usually modelled in Cartesian coordinates, whereas the target’s position and azimuth are obtained in polar coordinate. The radar is located at the origin, and provides range and bearing measurements. The measurement model can be established as

(40) $$z_{k}=\left(\begin{array}{l} \sqrt{x_{k}^{2}+y_{k}^{2}}\\ \mathrm{atan}2(y_{k},x_{k}) \end{array}\right)+v_{k}$$

where atan2(⋅) is the four-quadrant inverse tangent function, $v_{k}$ is the white Gaussian measurement noise with zero mean and covariance $R_{k}=diag([\sigma_{r}^{2},\sigma_{\theta }^{2}])$. $\sigma_{r}$ and $\sigma_{\theta }$ denote the standard deviation of range measurement noise and bearing angle measurement noise, respectively.

### 4.2. Simulation of the STSSRCKF

**Example** **1.** *In this simulation, the sampling interval is* T=1 s *and simulation time is* 100s*. The Monte Carlo simulations are carried out 200 times. The RMSE of the target position at time* k *and the accumulative RMSE (ARMSE) of estimated position at all times are defined in Equations (41) and (42):*

(41) $${\mathrm{RMSE}}_{\mathrm{pos}}(k)=\sqrt{\frac{1}{M}\sum_{m=1}^{M}\left({\left(x_{k}-{\hat{x}}_{m,k}\right)}^{2}+{\left(y_{k}-{\hat{y}}_{m,k}\right)}^{2}\right)}$$

(42) $${\mathrm{ARMSE}}_{\mathrm{pos}}=\sqrt{\frac{1}{N}\sum_{k=1}^{N}\left({\mathrm{RMSE}}_{\mathrm{pos}}^{2}(k)\right)}$$

*where* M *is the number of Monte Carlo runs,* $\left(x_{k},y_{k}\right)$ *is the actual value of the target position at time* k *and* $\left({\hat{x}}_{m,k},{\hat{y}}_{m,k}\right)$ *is the estimated position at time* k *in mth Monte-Carlo. The RMSE and the accumulative RMSE in the velocity and acceleration can be defined in the same way*.

This example considers a two-dimensional simulation scenario including one motion mode of high maneuver. The initial location of the target is (x,y)=(100 m,400 m), its initial velocity is $\left(v_{x},v_{y})=(15\;m/s,20\;m/s\right)$, and its initial acceleration is $\left(a_{x},a_{y})=(0{\;m/s}^{2},0{\;m/s}^{2}\right)$. The target makes a uniform motion during the first 150 s. Then, it takes a high maneuver with the acceleration $\left(a_{x},a_{y})=(15{\;m/s}^{2},25{\;m/s}^{2}\right)$ up to the end of this simulation at t=200 s. In this simulation, the initial value ${\hat{x}}_{0|0}$ and the initial covariance matrix $P_{0|0}$ are set to be $[100\;m,15\;m/s,{0\;m/s}^{2},$ 400 m, 20 m/s, $0{\;m/s}^{2}{]}^{T}$ and diag[ ${\left(50\;m\right)}^{2},$ ${\left(20\;m/s\right)}^{2},$ ${\left(1\;m/s^{2}\right)}^{2},$ ${\left(50\;m\right)}^{2},$ ${\left(10\;m/s\right)}^{2}$ ${\left(1\;m/s^{2}\right)}^{2}{]}^{T}$, respectively. The standard deviation of range measurement noise $\sigma_{r}$ is 30 m and the standard deviation of bearing angle measurement noise $\sigma_{\theta }$ is 10 mrad.

**Figure 1:**
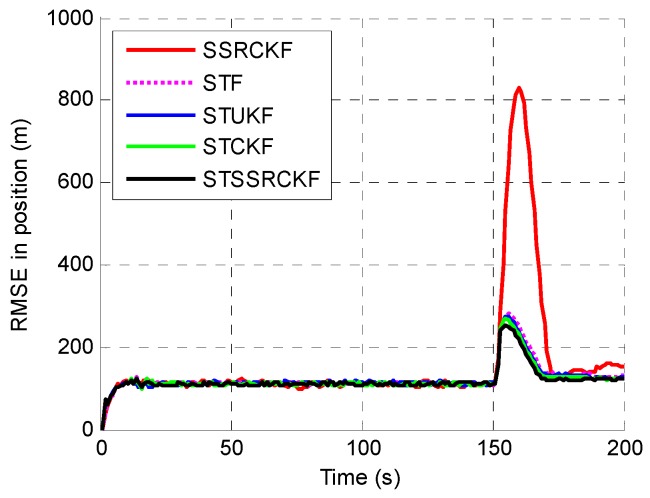
Root mean square error (RMSE) of the estimated position.

**Figure 2:**
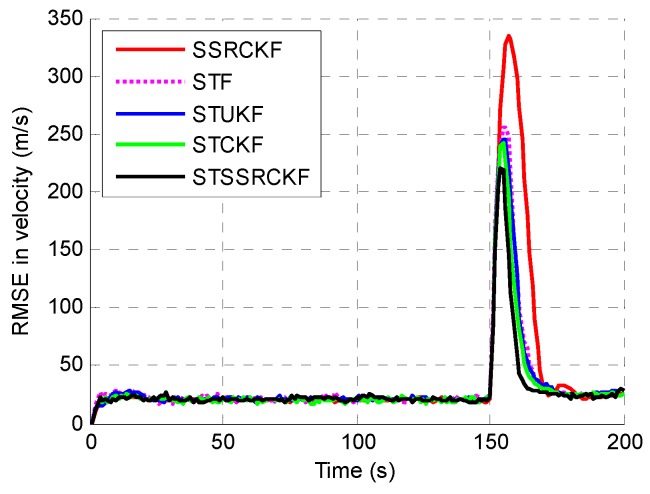
RMSE of the estimated velocity.

**Figure 3:**
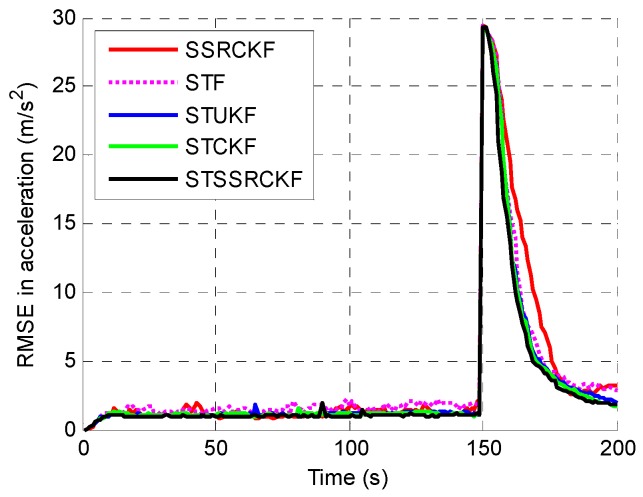
RMSE of the estimated acceleration.

The example is executed to examine the performance among the SSRCFK, STF, STUKF, STCKF and STSSRCK methods. The RMSEs of the position, velocity and acceleration using the five filters are shown in Figure 1, Figure 2 and Figure 3. It can be shown that the STF, STUKF, STCKF and STSSRCK methods can converge quickly when the target engages in high maneuvering. The SSRCKF algorithm only has a good performance for uniform motion. However, the performance of SSRCKF decreases seriously when the target engages in high maneuvering. This is because that the prediction covariance cannot be adjusted timely when the target state suddenly changes. The STF algorithm has the fourth speed of convergence, which is due to the fact that the linear approximation in the STF may introduce errors in the state which may lead the state to diverge. As can be seen from Figure 1, Figure 2 and Figure 3, when the target is making uniform motion within the first 100 s, the five methods have a similar performance. When the maneuver starts at t=101 s, it obviously shows that STF, STUKF, STCKF and STSSRCKF have the ability to convergence. The main reason is that the fading factor can adjust the prediction covariance and the corresponding filter gain in real time, which makes these algorithms converge in a short time. We can also see that the RMSE of the proposed algorithm is lower than that of STUKF and STCKF. It means that estimate precision of the proposed algorithm is higher than that of the two algorithms. It is demonstrated that the proposed algorithm can effectively track the abrupt motion state of the target.

To quantitatively describe the tracking performance, the ARMSEs of the five methods in estimating different target parameters are listed in Table 1. As shown, the STSSRCKF provided the best result in terms of estimation. The STCKF also performed well, followed by the STUKF and STF. The SSRCKF provided the worst estimate. We can also draw the conclusion that the STSSRCKF has the highest tracking accuracy of the position, velocity and acceleration.

The program is made on the Intel Core (TM) i5-4430 3.0GHZ CPU with 4.00G RAM. Table 2 shows the computational complexity and the computational time of SSRCFK, STF, STUKF, STCKF and STSSRCK for each run. Apart from STF, the computational complexity of different filters is mainly determined by the number of points they use. The computational complexity of STCKF as well as STUKF differs only by one points. The computational complexity of SSRCKF and STSSRCKF is *O*{(2*n* + 2)^3^}, where n denotes the dimension of state. In addition, we can see that the computational complexity of STF is the lowest. Because there is a clear formula in STF to calculate the Jacobian matrix, the computational complexity of STF is much smaller than other four algorithms. It is also shown that the computational time of the SSRCKF is 0.07 s for each run. However, the computational time of the STSSRCKF is 0.15 s, which is greater than that of the SSRCKF. This is because the STSSRCKF needs to calculate the suboptimal fading factor at each time step. At present, the time consumption is acceptable. The STSSRCKF needs more computational time than the SSRCKF, but considering the significant performance improvement gained from the STSSRCKF, this increased computational time is not substantial.

From this simulation, we can conclude that the STSSRCKF can perform the best in terms of the balance between computational complexity and estimation accuracy.

**Example** **2.** *This example evaluates the proposed algorithm in tracking a target with weak maneuver and medium maneuver. Therefore, two simulations are simulated as follows. Assume that there is a target making uniform at first. The initial location of the target is* (x,y)=(5000 m,5000 m)*, its initial velocity is* $\left(v_{x},v_{y})=(150\;m/s,80\;m/s\right)$*, and its initial acceleration is* $\left(a_{x},a_{y})=(0{\;m/s}^{2},0{\;m/s}^{2}\right)$*.*

Case 1: Simulation of medium maneuvering target tacking. The target moves with initial acceleration until t=150 s. Then, it maneuvers with acceleration of $(a_{x}(151),a_{y}(151))=$ $\left(5{\;m/s}^{2},5{\;m/s}^{2}\right)$ up to end of this simulation at t=200 s.
Case 2: Simulation of weak maneuvering target tracking. The initial position, velocity and acceleration of the target are the same as those in Case1. The target also moves with initial acceleration until t=150 s. Then, it maneuvers with acceleration of $\left(a_{x}(151),a_{y}(151))=(0.5{\;m/s}^{2},0.5{\;m/s}^{2}\right)$ up to end of this simulation at t=200 s.

Table 3 lists the accumulative RMSEs of the five methods in estimation the three target parameters. As can be seen from Table 3, the STSSRCKF algorithm also has a good tracking performance for a weak or medium maneuvering target.

## 5. Conclusions

To implement higher tracking accuracy for a maneuvering target, a new method has been proposed based on the STF and SSRCKF algorithms. Firstly, the time-varying suboptimal fading factor is introduced in order to adjust the prediction covariance and the corresponding filter gain in real time. Secondly, in the proposed method, the spherical simplex-cubature rule takes the place of calculating nonlinear function Jacobian matrix. In this way, STSSRCKF can converge rapidly in a short time. Thus, the proposed method has a high tracking accuracy for maneuvering target tracking. Simulation results show that the STSSRCKF can achieve higher accuracy and robustness than STF, STUKF, STCKF and SSRCKF, and indicate that it is suitable for maneuvering target tracking.

## Figures and Tables

**Table 1:** Tracking performance comparison.

Filters	Position ARMSE/m	Velocity ARMSE/(m/s)	Acceleration ARMSE/(m/s^2^)
SSRCKF	152.1	31.2	6.9
STF	129.7	28.4	6.2
STUKF	124.5	27.5	5.9
STCKF	123.1	26.7	5.8
STSSRCKF	119.3	25.1	5.6

**Table 2:** Computational complexity and computational time of different filters.

Filters	Computational Complexity	Computational Time (s)
SSRCKF	*O*{(2*n* + 2)^3^}	0.07
STF	*O*{(*n*)^2^}	0.02
STUKF	*O*{(2*n* + 1)^3^}	0.14
STCKF	*O*{(2*n*)^3^}	0.14
STSSRCKF	*O*{(2*n* + 2)^3^}	0.15

**Table 3:** ARMSEs in simulation of medium and weak maneuvering target.

Simulation	Filters	Position ARMSE/m	Velocity ARMSE/(m/s)	Acceleration ARMSE/(m/s^2^)
Case 1	SSRCKF	101.6	21.2	5
	STF	95.3	20.2	4.5
	STUKF	88.5	16.4	4.1
	STCKF	87.4	16.8	4.1
	STSSRCKF	81.1	15.9	3.7
Case 2	SSRCKF	50.5	8.2	1.8
	STF	65.1	10.8	2.4
	STUKF	57.3	8.8	2.2
	STCKF	56.3	8.3	2.2
	STSSRCKF	53.4	8.4	2.1
